# Paraspeckle Protein 1 (PSPC1) Is Involved in the Cisplatin Induced DNA Damage Response—Role in G1/S Checkpoint

**DOI:** 10.1371/journal.pone.0097174

**Published:** 2014-05-12

**Authors:** Xiangjing Gao, Liya Kong, Xianghong Lu, Guanglin Zhang, Linfeng Chi, Ying Jiang, Yihua Wu, Chunlan Yan, Penelope Duerksen-Hughes, Xinqiang Zhu, Jun Yang

**Affiliations:** 1 Collaborative Innovation Center for Diagnosis and Treatment of Infectious Diseases, The First Affiliated Hospital, Zhejiang University, Hangzhou, Zhejiang, China; 2 Department of Toxicology, Zhejiang University School of Public Health, Hangzhou, Zhejiang, China; 3 Department of preventative medicine, Zhejiang Chinese Medical University, Hangzhou, China; 4 Lishui People's Hospital, Lishui, Zhejiang, China; 5 Center Testing International Corporation, Shenzhen, Guangdong, China; 6 Department of Basic Science, Loma Linda University School of Medicine, Loma Linda, Californina, United States of America; 7 Department of Toxicology, Hangzhou Normal University School of Public Health, Hangzhou, Zhejiang, China; 8 Department of Biomedicine, College of Biotechnology, Zhejiang Agriculture and Forestry University, Hangzhou, China; Universita' di Milano, Italy

## Abstract

Paraspeckle protein 1 (PSPC1) was first identified as a structural protein of the subnuclear structure termed paraspeckle. However, the exact physiological functions of PSPC1 are still largely unknown. Previously, using a proteomic approach, we have shown that exposure to cisplatin can induce PSPC1 expression in HeLa cells, indicating the possible involvement for PSPC1 in the DNA damage response (DDR). In the current study, the role of PSPC1 in DDR was examined. First, it was found that cisplatin treatment could indeed induce the expression of PSPC1 protein. Abolishing PSPC1 expression by siRNA significantly inhibited cell growth, caused spontaneous cell death, and increased DNA damage. However, PSPC1 did not co-localize with γH2AX, 53BP1, or Rad51, indicating no direct involvement in DNA repair pathways mediated by these molecules. Interestingly, knockdown of PSPC1 disrupted the normal cell cycle distribution, with more cells entering the G2/M phase. Furthermore, while cisplatin induced G1/S arrest in HeLa cells, knockdown of PSPC1 caused cells to escape the G1/S checkpoint and enter mitosis, and resulted in more cell death. Taken together, these observations indicate a new role for PSPC1 in maintaining genome integrity during the DDR, particularly in the G1/S checkpoint.

## Introduction

Cells are continuously faced with exogenous and endogenous stress that can induce DNA damage, potentially leading to genomic instability and cell death [1]. To maintain genomic integrity, cells have evolved the DNA damage response (DDR), a complex network of interacting pathways. Usually, DNA damage is primarily detected by the MRE11–RAD50–NBS1 (MRN) complex, which is followed by the activation of the phosphatidylinositol 3-kinase-like protein kinase (PIKKs) family members: ataxia telangiectasia mutated protein (ATM), ataxia telangiectasia and Rad3-related protein (ATR) and DNA dependent protein kinase (DNA-PK) [2]–[4]. These kinases phosphorylate and activate a variety of substrates to execute various cellular functions such as DNA repair, cell cycle arrest and cell death. One substrate is the histone variant H2AX, which can be phosphorylated at Ser-139 (termed γH2AX) and is directly involved in DNA repair [5], [6]. Phosphorylation of H2AX is required to recruit a number of DDR proteins including repair factors and chromatin remodeling complexes [7]–[9]. For this reason, γH2AX foci formation has been recognized as an effective indicator of DNA damage, even when only a few DNA double-strand breaks (DSBs) are elicited [10]–[12]. As a mediator/adaptor of DDR, 53BP1 can facilitate ATM-dependent phosphorylation events, including the efficient phosphorylation of checkpoint kinase 2 (CHK2), and is required for ATM-dependent repair of DSBs through the non-homologous end-joining (NHEJ) pathway [13]–[15]. Similarly, in the homologous recombination (HR) pathway, the Rad51 protein interacts with the ssDNA-binding protein (SSBs) and re-localizes with the nucleus to form distinct foci, which represent repair active sites [16]. It is well known that proteins involved in DNA repair usually, either bind directly to the DNA at a damaged site such as Ku and Rad52 proteins [17], [18], or interact with other repair proteins as part of the repair complex at the damaged site (referred as the “repair foci”) [19]. These proteins, together with many other DNA repair proteins, are important in maintaining genome stability. As would be expected, defective DNA damage repair is associated with various developmental, immunological, and neurological disorders, and is a major driver in cancer [20].

During DDR, cell cycle checkpoints, including the G1/S and G2/M checkpoints, can be activated before replication or mitosis ensues, respectively [21], [22]. Cells can arrest the cell cycle temporarily to allow for: (i) cellular damage to be repaired; (ii) the dissipation of an exogenous cellular stress signal; or (iii) availability of essential growth factors, hormones or nutrients [23], [24]. If the damage can be effectively repaired during cell cycle, cells can regain normal functions and resume the cell cycle. Alternatively, if cell cycle checkpoint fails and the damage cannot be successfully repaired, chronic DDR can trigger cell death through mechanisms such as apoptosis or cellular senescence [25], [26]. The checkpoint response, which prevents cells from accumulating mutations through replication and possibly developing into cancer, is a critical part of the DDR [27], [28].

Because of the importance of DDR in cell growth and survival, numerous studies have been conducted to identify the many proteins/molecules involved and to reveal the underlying mechanisms. High-throughput technologies, such as genomics and proteomics, can generate huge amounts of information, and data mining of this information can reveal previously unknown or unexpected associations. Therefore, such technologies are useful tools for identifying new molecules/pathways involved in cellular activities such as DDR. Previously, using such an approach, e.g., nuclear proteomics, we investigated the induction of DDR in HeLa cells by cisplatin, a first-line chemotherapeutic agent with DNA damaging properties. Interestingly, among the many proteins affected by cisplatin treatment, we found that the expression of paraspeckle protein 1 (PSPC1) could be induced by cisplatin, suggesting it as a newly-discovered participant in cisplatin-induced DDR [29].

PSPC1 was first identified as a structural protein of a specific type of nuclear body called the paraspeckle [30]. Paraspeckles are involved in transcriptional and post-transcriptional gene regulatory functions, such as controlling expression of hyper edited mRNAs, mRNA biogenesis, pre-mRNA 3′-end formation, cyclic AMP signaling, and nuclear receptor-dependent transcriptional regulation [31]–[33]. PSPC1 contains two copies of the RNA recognition motif (RRM), which is the most prevalent RNA-binding domain in eukaryotes and a prerequisite for the localization of PSPC1 to paraspeckles. Another two proteins, polypyrimidine tract-binding protein associated splicing factor (PSF) and 54 kDa nuclear RNA binding protein (p54nrb) contain two RRMs and together with PSPC1, comprise the protein core of paraspeckles in HeLa cells. In addition to their functional role in the paraspeckle, previous studies also showed their role in cell survival or proliferation. For example, it was shown that attenuating p54nrb expression in human colon cancer HCT-116 cells resulted in smaller colony size and lower plating efficiency [34], but knockdown of p54nrb had no effect on long-term survival in HeLa cells [35]. PSF knockdown severely inhibited cell proliferation in DLD-1 cells [36], and caused a more severe loss of cell viability in the Rad51D-deficient mouse embryonic fibroblast (MEF) cells than in the corresponding Rad51D-proficient cells [37]. Also, it has been shown that PSF and p54nrb form a stable complex *in vivo*, which is involved in the repair of DSBs *via* the HR pathway [34], [38]. Furthermore, the PSF·p54nrb complex is involved in NHEJ in vertebrates [39], [40].

In contrast, the functions of PSPC1 are largely unknown with the exception of its possible involvement in regulating either gene expression or RNA processing. For example, Myojin *et al* showed that PSPC1 has RNA-binding activity [41], and Fox *et al* reported that PSPC1 might be involved in the regulation of mRNA splicing [42]. Other studies suggested that PSPC1 might regulate androgen receptor-mediated transcriptional activity [43]. Interestingly, one earlier study, which analyzed ATM and ATR substrates in an effort to reveal the extensive protein network activated in response to DNA damage, identified PSPC1 as a possible phosphorylation substrate of ATM/ATR [44]. Furthermore, Ha *et al* reported that PSF could promote the recruitment of PSPC1 to sites of DNA damage following knockdown of p54nrb [40]. Such information, combined with our observation that PSPC1 expression can be induced by cisplatin as well as evidence that the other two paraspeckle proteins, PSF and p54nrb, are involved in DNA repair, all lead to the hypothesis that PSPC1 is very likely a participant in the DDR. However, the precise role of PSPC1 in DDR has not yet been carefully investigated. To address this question, we carried out a series of analyses designed to reveal a possible role of PSPC1 in the DDR, and as reported here, we provide the first piece of evidence for the direct involvement of PSPC1 in DDR. Specifically, we provide evidence for its function at the G1/S checkpoint.

## Methods

### Cell culture and cell cycle synchronization

Human cervical carcinoma (HeLa) cells obtained from the ATCC were grown in Minimal Essential Medium (MEM) supplemented with 10% new born calf serum (NCS) with 5% CO_2_ at 37°C. Cell cycle synchronization was carried out by double thymidine blockage at the G1/S boundary as described in [45]. Briefly, cells were grown in the presence of 2 mM thymidine (Sigma, St. Louis, MO) for 18 h, then washed with PBS, and grown in fresh medium without thymidine for 8 h. Thymidine was added again at 2 mM and incubated another 18 h to block cells at the G1/S boundary.

### Chemicals and antibodies

Cisplatin was purchased from Sigma; PSF and p54nrb antibodies were purchased from Santa Cruz Biotechnology (Santa Cruz, CA), mouse monoclonal anti-β-actin antibody and the Annexin V-fluoresce isothiocyanate (FITC)/propidium iodide (PI) apoptosis detection kit were obtained from Multisciences Biotechnology (Hangzhou, China). γH2AX, Rad51 and 53BP1 antibodies were purchased from Millipore (Billerica, MA); Caspase-3 and PARP antibodies were supplied by Bioworld Technology (St. Louis Park, MN); and an affinity-purified peptide antibody against PSPC1 was generated in rabbits in our laboratory as described by Fox *et al* [42]. Alexa Fluor 488-conjugated and IR Dye-conjugated goat anti-mouse and goat anti-rabbit IgG were obtained from Life Technologies (Carlsbad, CA, USA).

### Transfection of small interfering RNA (siRNA) and detection of PSPC1 expression

Two sets of siRNA oligo nucleotides for the human PSPC1 gene corresponding to nucleotides 1257—1275 (siPSPC1) and negative control siRNA were synthesized by Shanghai GenePharma Co., Ltd and used for transfection. siRNAs were transfected into HeLa cells using Lipofectamine2000 (Invitrogen, Carlsbad, CA), essentially as directed by the manufacturer and using a siRNA concentration of 40 nM. In short, cells were seeded into a 6-well cell culture plate, siRNA-Lipofectamine2000 complexes were added to each well after 24 h, and the medium was changed after 6 h incubation. After 18 h incubation, the attenuation of mRNA levels was detected by real-time reverse transcriptase PCR (RT-PCR). Total RNA was isolated using Trizol Reagent (Invitrogen), and 2 µg of total RNA was used for first-strand cDNA synthesis with Super Script III Reverse Transcriptase (Invitrogen). RT-PCR was performed in 20 µl using the TakaRa SYBR *Premix Ex Taq* Kit (TaKaRa Biotechnology, Dalian, China) and 100 ng of input cDNA template. β-actin was used as an internal standard. Primers for PSPC1 were 5′-AGACGCTTGGAAGAACTCAGA-3′ and 5′-TTGGAGGAGGACCTTGGTTAC-3′; primers for β-actin were 5′-TGCGTGACATTAAGGAGAA-3′ and 5′-AAGGAAGGC TGGAAGAGT-3′.

### Plasmid vectors and transfection

The pPSPC1 and pCON plasmids were constructed by Shanghai Genechem Co., Ltd (G006). Cells were transfected with 2 µg plasmid as well as the empty vector in Opti-MEM medium (Invitrogen) with X-tremeGENE HP DNA transfection reagent (Roche) according to the manufacturer's protocol.

### Immunoblotting

Cells were lysed in RIPA lysis buffer (Beyotime, Nantong, China), and protein concentrations were determined using the bicinchoninic acid (BCA) Protein Assay Kit (Beyotime). Denatured protein extracts were loaded and separated on 15% or 8% SDS–polyacrylamide gels (Mini-Protean II, Bio-Rad) and transferred to an Immunoblot polyvinylidene fluoride (PVDF) Membrane (Millipore). After blocking with 3% non-fat milk in Tris-buffed saline with 0.1% (v/v) Tween-20 (TBST), membranes were incubated with primary antibodies at 4°C overnight, followed by incubation of IR Dye-conjugated secondary antibodies for 1 h at room temperature. After three washes, membrane-bound proteins of interest were detected using an Odyssey Infrared Imaging System (Li-Cor, USA).

### Assessment of cell viability

Cell viability was determined using the Trypan blue exclusion assay as described previously [46]. In short, cells were treated with trypsin, removed from the plate and centrifuged for 5 min at 250 g. The pellet was suspended in MEM. Equal volumes of 0.4% Trypan blue and the cell suspension were mixed and 10 µl of the mixture was applied to a hemocytometer. The stained (non-viable) and unstained (viable) cells were counted under a microscope.

### Analysis of apoptosis

The Annexin V-FITC/PI kit (Multiscience) was used to analyze the extent of apoptosis. Briefly, cells were collected by trypsinization and washed three times with phosphate-buffered saline (PBS), then resuspended in 500 µl binding buffer with 5 µl Annexin V-FITC and 10 µl PI. Cells were incubated for 5 min in the dark at room temperature. The cells were then analyzed using a FC500 MCL machine (Beckman Coulter) at 10,000 events/sample.

### Immunofluorescence microscopy

For immunofluorescent staining, cells were fixed in 4% paraformaldehyde for 15 min, permeabilized with 0.5% triton and blocked with 3% BSA for 1 h at 37°C. The cells were incubated with primary antibodies overnight, washed three times in PBS, and then incubated with Alexa Fluor 488-conjugated secondary antibodies for 1 h. DNA was counterstained with 1 µg/ml DAPI for 15 min at 37°C. Cells mounted on cover slips were observed with a Leica DMI 4000 immunofluorescent microscope or a Zeiss confocal laser scanning microscope.

### Cell cycle analysis

For flow cytometry measurements of the cell cycle, 36 h-post transfection cells were trypsinized, centrifuged at 300 g for 5 min and fixed overnight in 70% cold ethanol at −20°C. After washing twice with PBS, the cells were resuspended in 500 µl of fresh PBS containing 50 µl of 2 mg/ml RNaseA and 10 µl of 1 mg/ml PI (Sigma). Cells were incubated for 15 min at 37°C. The cells were then analyzed immediately using a FC500 MCL machine (Beckman Coulter) at 10,000 events/sample.

### Statistical analysis

Statistical analysis was performed using the Student's t-test or one-way ANOVA. Each experiment was conducted at least three times independently. Data were presented as mean ± SD and a probability level of *P*< 0.05 was considered significant.

## Results

### PSPC1 expression in HeLa cells is induced by cisplatin

**Figure 1:**
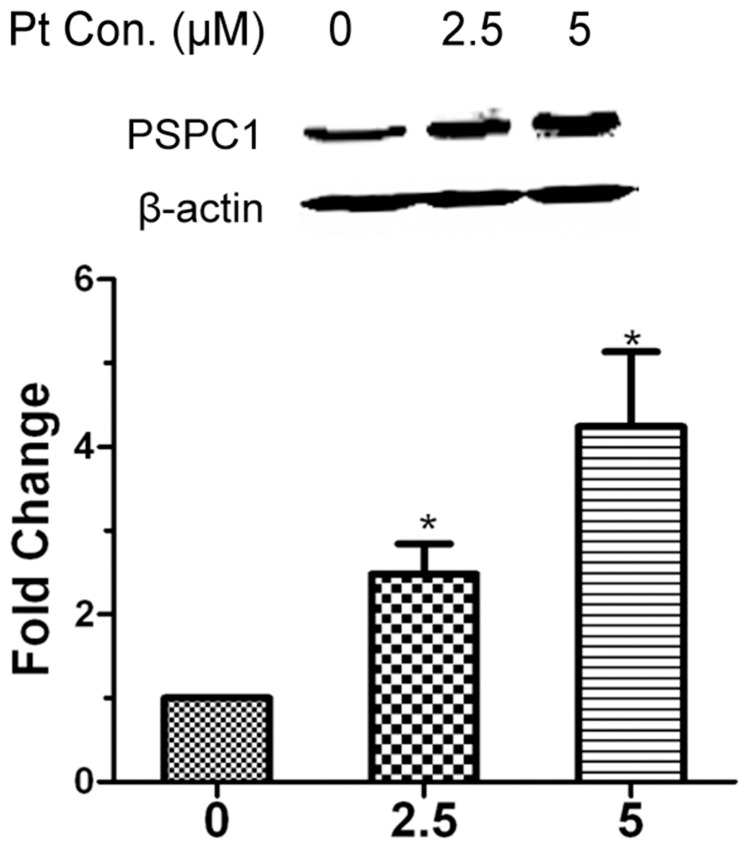
PSPC1 is induced by cisplatin. HeLa cells were treated with 2.5 or 5 µM of cisplatin (Pt) for 12 h, and expression of PSPC1 was detected by Western blot. The results are shown as the mean ±SD of three independent experiments. *P<0.05, compared with the control group.

Previously, we had employed nuclear proteome analysis to demonstrate that PSPC1 could be induced by cisplatin in HeLa cells [29]. To further validate this observation, HeLa cells were treated with different doses of cisplatin for 12 h, and the expression of PSPC1 was examined by Western blot. As shown in Figure 1, the level of PSPC1 was indeed increased by cisplatin treatment. Cisplatin concentrations at 10 µM or higher were not examined as significant loss of cell viability was induced (data not shown). Therefore, all the following experiments using cisplatin were conducted at concentrations of either 2.5 or 5 µM.

### Knockdown of PSPC1 reduces cell survival

**Figure 2:**
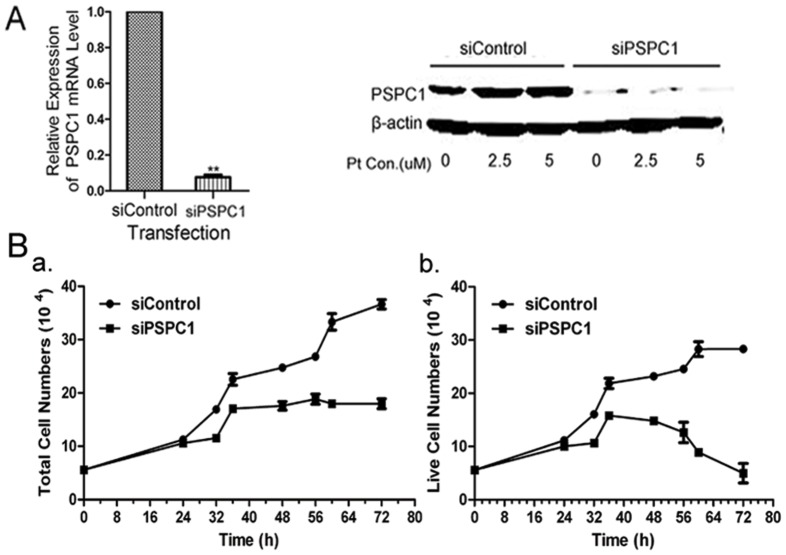
Attenuation of PSPC1 expression inhibits cell proliferation. (A) HeLa cells were transfected with 40 nM PSPC1 siRNAs (siPSPC1) or control siRNA (siControl) (‘Materials and Methods’ section). 24 h later, expression of PSPC1 was analyzed using quantitative real-time PCR (left histogram) and Western blot (right panels). β-actin was used as the loading control. (B) Cell proliferation of HeLa cells transfected with siPSPC1 or siControl was measured by the Trypan blue exclusion assay. Left, total cell number; Right, viable cell number. Data represents the average of three independent experiments with six replicate measurements (mean ± SD).

**Figure 3:**
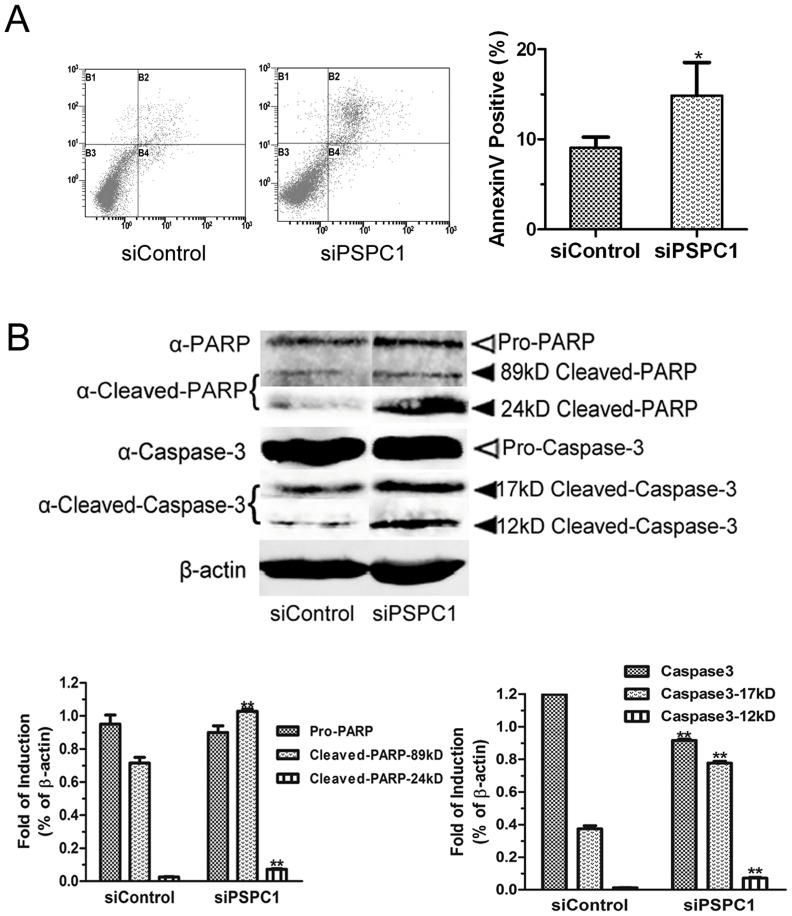
Knockdown of PSPC1 induces cell death. (A) HeLa cells harvested at 24 h post-transfection were analyzed by dual-parameter flow cytometry utilizing Annexin V-FITC and PI. Representative dot plot data from three independent experiments are shown in the left panel, and the histogram graph at right represents the percentage of dual-parameter positive cells pooled from three independent experiments. (B) HeLa cells harvested at 24 h post-transfection were analyzed by Western blotting to evaluate the expression of Caspase-3 and PARP. Densitometric data of three independent experiments are presented below the immunoblot, and β-actin was used as an internal standard. Data are presented as mean ± SD. *P< 0.05, **P< 0.01, compared with control group.

To explore the possible biological functions of PSPC1, we first examined the effects of PSPC1 siRNA knockdown on cell growth and cell death. Transfection with PSPC1 siRNA consistently reduced mRNA and protein expression by about 95% compared with control siRNA, as assessed by both RT-PCR and Western blot (Figure 2A). Trypan blue exclusion assay results showed that PSPC1 knockdown significantly inhibited cell growth (Figure 2B, left panel). Furthermore, although there was a slight increase at early hours (up to 36 h), the number of live cells then gradually decreased, eventually dropping to less than the originally seeded number of cells by 72 h in the siPSPC1 group (Figure 2B, right panel). This observation implies an important role for PSPC1 in maintaining cell viability. Therefore, we further evaluated the effects of PSPC1 on cell death. As shown in Figure 3A, about 10% of the cells were Annexin V and PI-positive in the control group, in contrast, after PSPC1 knockdown, the percentage of dual-positive cells was 15%, a slight but significant increase. In addition, we also assessed the level of cleaved Caspase-3 and cleaved PARP by Western blot, which are considered markers of apoptosis. As shown in Figure 3B, cleaved Caspase-3 and cleaved PARP were significantly up-regulated after knockdown of PSPC1 in HeLa cells, suggesting that some of the PSPC1-knockdown cells undergo apoptosis by caspase and/or PARP-dependent mechanisms.

### Alteration of PSPC1 expression influences the formation of γH2AX foci

**Figure 4:**
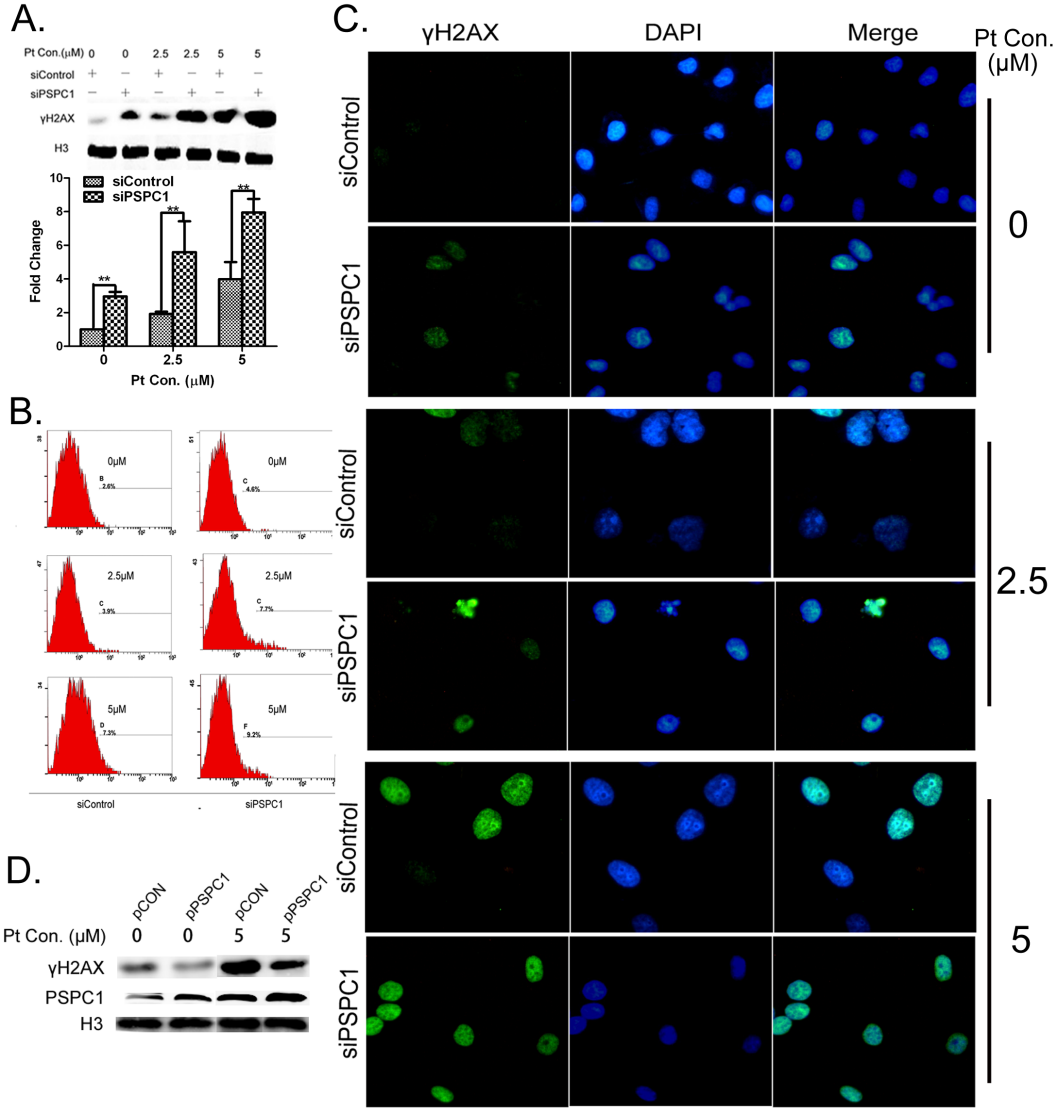
Alteration of PSPC1 expression influences the formation of γH2AX foci. HeLa cells were transfected with siPSPC1 or siControl. 24-transfection, cells were treated with 2.5 or 5 µM of cisplatin for 12 h, and the expression of γH2AX was examined by Western blot (A), flow cytometry (B), and immunofluorescence microscopy (C). (D) HeLa cells were transfected with either pPSPC1 or pCON to overexpress PSPC1. 24 h post-transfection, cells were treated with 5 µM of cisplatin for 12 h, and the expression of γH2AX or PSPC1 was examined by Western blot. *P< 0.05, compared with control.

As our interest was the possible role of PSPC1 in DDR, we then measured the extent of cisplatin-induced DNA damage in the presence or absence of PSPC1 using γH2AX foci formation as a sensitive indicator. Interestingly, Western blot data showed that PSPC1 knockdown resulted in a marked increase in the level of γH2AX in cells even without cisplatin exposure (Figure 4A). Cisplatin treatment induced a dose-dependent increase in γH2AX protein levels, and the level of this increase was much stronger in each siPSPC1 group as compared with the corresponding siControl group (Figure 4A). Flow cytometry and immunofluorescence results demonstrated the same trend (Figure 4B and 4C).

To further verify whether PSPC1 expression can influence cisplatin-induced DNA damage, HeLa cells were transfected with an overexpression plasmid of PSPC1. As shown in Figure 4D, overexpression of PSPC1 in HeLa cells significantly inhibited the increase of γH2AX protein level compared to control cells, implying less severe DNA damage. Together, these findings suggested that PSPC1 is important in maintaining DNA stability and minimizing genomic insults in cells.

### PSPC1 does not form distinct foci with γH2AX, 53BP1 nor Rad51

**Figure 5:**
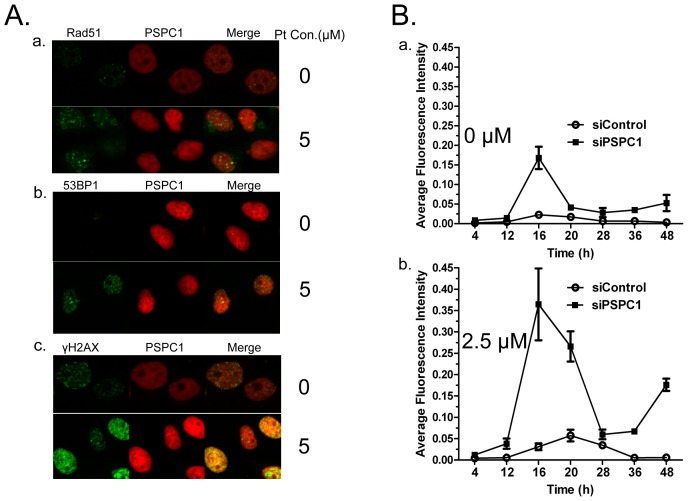
PSPC1 may not participate in DNA repair. (A) PSPC1 does not form distinct foci with γH2AX, 53BP1 or Rad51. Representative confocal laser scanning images of HeLa cells were analyzed 12 h after 5 µM cisplatin treatment. (B) DNA repair kinetic curve in siControl and siPSPC1 cells as calculated by the intensity of γH2AX measured by immunofluorescence microscopy. Quantitative analysis of average density (Fluorescence intensity per unit area) was determined by Image-Pro Plus 6.0.

As noted above, cisplatin can induce increased expression of PSPC1 (Figure 1), and the loss of PSPC1 results in increased DNA damage (Figure 3). Therefore, it is reasonable to predict that PSPC1 might play a role in DNA repair and in this way protect cells from cisplatin-induced damage. To investigate this possibility, we examined the distribution of PSPC1, as well as its relationship with several key factors involved in DNA repair, including γH2AX, 53BP1, and Rad51. The results (Figure 5A) showed that there were no significant changes in the relatively diffuse distribution pattern of PSPC1 in the nucleus in both control and cisplatin treated cells. In contrast, cisplatin induced the formation of distinct Rad51, 53BP1 and γH2AX foci as compared with their respective controls. In addition, upon close examination, PSPC1 did not co-localize with Rad51, 53BP1, or γH2AX to form distinct foci after cisplatin treatment (Figure 5A). Taken together, these results fail to support the idea that PSPC1 participates in the specific DNA repair events mediated by Rad51, 53BP1 and γH2AX.

Studies of the DNA repair function of p54nrb showed that knockdown of p54nrb could lead to a delay in the repair of DNA damage [34]. This suggested an alternate mechanism for PSPC1 action, and to further examine the possible DNA repair activity of PSPC1, we measured the level of γH2AX during a 48 h period as an indicator of DNA repair in the presence and absence of PSPC1. The results showed that in control siRNA cells, the γH2AX foci level remained low, as expected. In contrast, knockdown of PSPC1 with siRNA led to a burst of γH2AX formation at 16 h. These lesions were repaired rapidly, and the level of γH2AX decreased to a level slightly higher than that of control cells after 20 h (Figure 5B, top panel). Following cisplatin treatment, the increase in γH2AX foci appeared earlier in PSPC1 knockdown cells than in the control cells. These cells also showed a burst in γH2AX formation at about 16 h, followed by the rapid repair, although γH2AX level remained higher than in control cells (Figure 5B, lower panel). Therefore, although the repair kinetic curve is quite different in the presence and absence of PSPC1, there is no clear delay of repair in PSPC1-knockdown cells as compared with control cells.

### Loss of PSPC1 causes cells to enter G2/M phase

**Figure 6:**
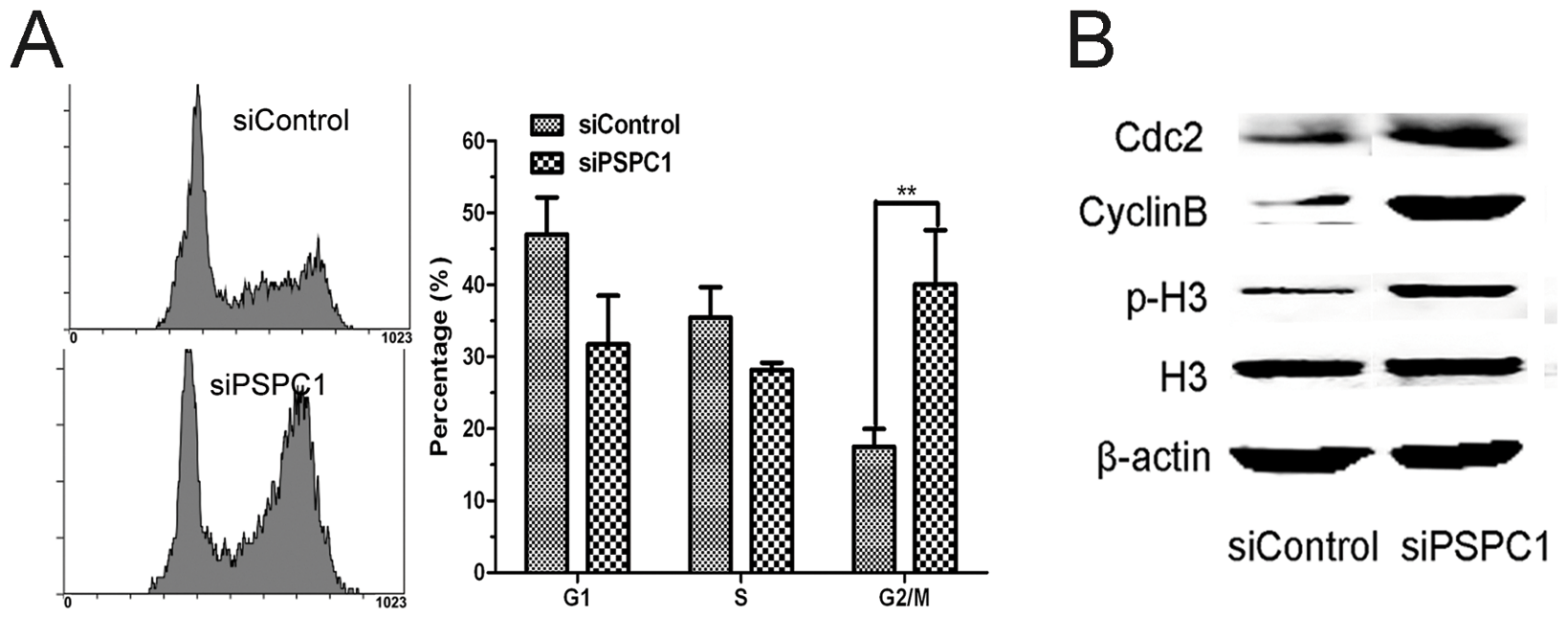
Loss of PSPC1 causes cells to enter G2/M phase. (A) HeLa cells were cultured in the presence of 2 mM thymidine for 18 h, washed with PBS, and transfected with siRNAs using Lipofectamine2000. 8 h after transfection, thymidine was added again to 2 mM to block cells at the G1/S boundary. After another 18 h, cells were transferred to fresh medium for 24 h, then harvested and analyzed by flow cytometry. (B) Using the same cells, the levels of representative G2/M phase proteins (phospho-histone H3 (Ser10), Cdc2 and cyclinB [55]) were examined by Western blot.

Upon DNA damage, mammalian cells may activate cell-cycle arrest to stop or delay cell division to allow the damage to be repaired [47]. As the above results did not support a direct role for PSPC1 in DNA repair, we asked whether PSPC1 might function in cell cycle progression. siPSPC1 or siControl-transfected HeLa cells were first synchronized at the S phase, then allowed to grow in fresh medium for 24 h, and subjected to cell cycle analysis. The results showed that for control siRNA transfected cells, 48% of the cells were in G1, 35% in S, and 17% in the G2/M phase; however, for siPSPC1 cells, the ratio was: 35% in G1, 27% in S, and 38% in the G2/M, a more than 2-fold increase in the number of cells entering G2/M (Figure 6A).

To confirm whether these cells were indeed entering the G2/M phase, the expression levels of phospho-histone H3, cyclinB and Cdc2, known regulatory proteins of the G2/M phase [48], were measured by Western blot. As shown in Figure 6B, PSPC1 knockdown markedly increased the level of phospho-histone H3. Similarly, the levels of cyclinB and Cdc2 were also increased significantly after attenuation of PSPC1 expression (Figure 6B). Therefore, these data pointed out a possible involvement for PSPC1 in regulating the cell cycle.

### PSPC1 is involved inG1/S phase arrest induced by cisplatin

**Figure 7:**
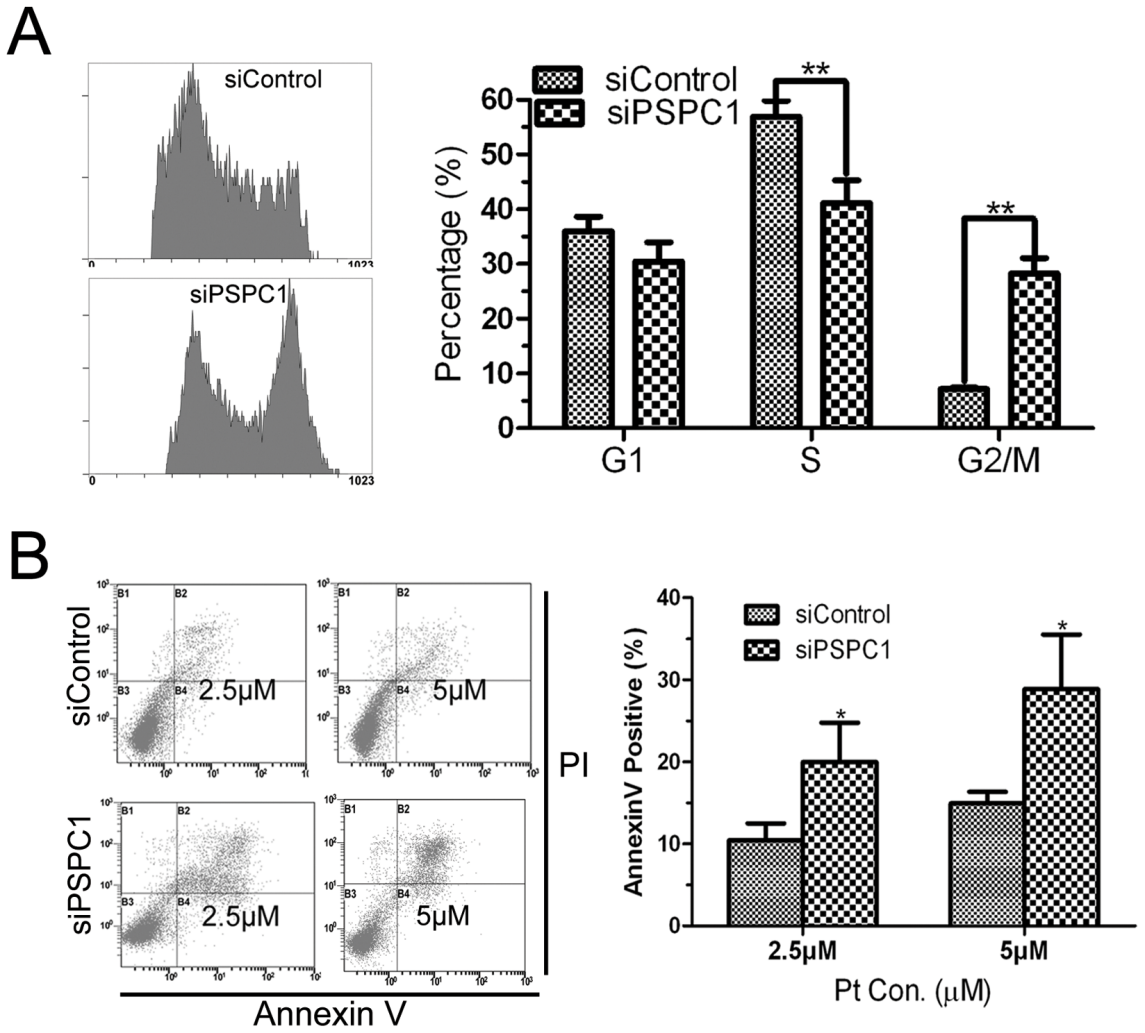
Knockdown of PSPC1 causes cells to escape cisplatin-induced G1/S arrest. (A) HeLa cells were synchronized by double thymidine blockage at the G1/S boundary as described previously, released into fresh medium with 5 µM cisplatin for 24 h, and then harvested and detected by flow cytometry for cell cycle distribution. (B) The same synchronized HeLa cells were treated with 2.5 or 5 µM of cisplatin for 24 h, then apoptosis was analyzed by dual-parameter flow cytometry with Annexin V-FITC and PI staining. Representative dot plot data from three independent experiments are shown left, and the right histogram represents the percentage of non-viable cells pooled from three independent experiments. *P< 0.05, **P< 0.01, compared with control group.

To further clarify the function of PSPC1 in cell cycle regulation, we examined the cell cycle distribution of HeLa cells upon cisplatin exposure. Cisplatin treatment is known to induce S arrest in cells [49], and our results showed that after 24 h of cisplatin treatment, siControl exhibited a clear S phase arrest, with about 57% cells in S phase (Figure 7A, compared to 35% in Figure 6A) and 8% in the G2/M phase. On the other hand, knockdown of PSPC1 by siRNA attenuated the cisplatin-induced S phase-arrest, with only 42% in S phase but about 30% of cells in the G2/M phase, almost a 4-fold increase compared with the siControl cells (Figure 7A).

In addition, cisplatin-induced cell death was also measured. As shown in Figure 7B, compared with siControl, the percentage of dead cells was significantly increased following PSPC1 siRNA knockdown (∼2-fold). Taken together, these data suggest that PSPC1 might play a role in regulation of the G1/S checkpoint, whereas disruption of its function could lead to cells escaping the G1/S arrest and entering G2/M phase. These events have the potential to increase DNA damage and to cause more cell death.

## Discussion

PSPC1 is found in paraspeckles in transcriptionally active cells as well as perinucleolar caps in cells that are not actively transcribing Pol II genes [42]. It belongs to the Drosophila Behavior Human Splicing (DBHS) family, which is composed of two classical RRMs followed by a proline-rich coiled-coil motif [32]. Together with other two DBHS family members, PSF and p54nrb, PSPC1 forms the protein core of paraspeckles. To date, many studies have been conducted to investigate the functions of PSF and p54nrb, and the results pointed out their important roles in DNA repair. In contrast, PSPC1 is more selectively expressed, acting as a coactivator of transcription [43]. It is also known that PSPC1 can dimerize with p54nrb through the coiled-coil domain to regulate pre-RNA processing, but not with PSF [50]. Nevertheless, until now, little is known about its other functions, especially in DDR.

Previously, as part of an effort to investigate the DDR, we conducted a nuclear proteomics screen for DDR-related proteins. This screen identified PSPC1 as a novel molecule possibly participating in cisplatin-induced DDR [29]. Combined with previous reports stating that (i) PSPC1 could be phosphorylated by ATM/ATR [44], (ii) p54nrb and PSF are involved in DSB repair, and (iii) PSF could promote the recruitment of PSPC1 to sites of DNA damage after p54nrb knockdown [40], the involvement of PSPC1 in DDR seemed a reasonable possibility. To test this hypothesis, we first showed that PSPC1 could indeed be induced by cisplatin (Figure 1). This phenomenon is characteristic for proteins participating in the DDR, for example, p53 and proline-rich acidic protein 1 (PRAP1), key regulators of DDR, can be induced under conditions of DNA damage [51], [52]. Thus, this is the first piece of evidence linking PSPC1 to DDR. To clarify the physiological function of PSPC1, we then inhibited the expression of PSPC1 by siRNA, and examined the effects of this knockdown on cell growth and survival. These results showed that depletion of PSPC1 significantly inhibited cell proliferation (Figure 2). The effects of knocking down either PSF or p54nrb on cell survival or proliferation have been previously investigated by others. Those studies indicate that the effects of these proteins on cell proliferation are likely to be cell-type specific due to different genetic backgrounds. Nonetheless, our data indicated an important role for PSPC1 in maintaining normal cell growth, at least in HeLa cells.

Additionally, our results showed that after loss of PSPC1, the number of live cells was dramatically reduced (Figure 2), indicating the occurrence of cell death. The activation of Caspase-3 and PARP further demonstrated that knockdown of PSPC1 indeed can cause apoptosis (Figure 3). Similarly, PSF knockdown also induced Caspase-3 mediated apoptosis in DLD-1 cells [36], suggesting that PSPC1 and PSF might share certain common functions. However, it should be noted that the loss of PSPC1 increased the number of apoptotic cells only to a small extent, while the number of live cells decreased rather dramatically. Thus, it is believed that other types of cell death, including necrosis, autophagy, or necroptosis may also be occurring, and is an area of ongoing and future study.

As our focus is the relationship between PSPC1 and DDR, we next evaluated the effects of PSPC1 knockdown on cisplatin-induced DNA damage. Our results showed that depletion of PSPC1 sensitized cells to cisplatin-induced DNA damage, as assessed by the appearance of γH2AX foci (Figure 4). This is consistent with previous reports indicating that knocking down p54nrb sensitized cells to radiation [34]. Such similarity provides another piece of evidence for the involvement of PSPC1 in DDR.

As PSF and p54nrb play important roles in DNA repair during DDR, we were wondering whether PSPC1 also had a similar function. Previously, PSF and p54nrb have been shown to bind directly to DNA, and can interact with other repair proteins such as Rad51 [35], [53]. For this reason, we used confocal microscopy to ask whether PSPC1 could form foci upon cisplatin exposure, and whether it interacted with other repair proteins. Unexpectedly, we did not observe the formation of distinct PSPC1 foci following cisplatin treatment. Furthermore, the fluorescent image did not support a co-localization of PSPC1 with any of the three DDR tested proteins (Figure 5A), thus casting doubt on the idea that PSPC1 participates in DNA repair through direct interactions with these proteins. We then reasoned that if a protein is involved in DNA repair, disruption of its function would be expected to lead to the delay of DNA repair, as in the case for p54nrb [34], [35]. Thus, we monitored DNA repair in PSPC1 knockdown cells using the disappearance of γH2AX as an indicator. As shown in Figure 5B, although the extent of DNA damage was much severe in PSPC1 knockdown cells, the rate of repair was almost the same as in control cells. Together, these data implied that PSPC1 might not function in DNA repair, a situation that is quite different from that seen for PSF and p54nrb.

If PSPC1 were not involved in DNA repair, then what was its role in the DDR? One thing we noticed is that in the DNA repair curve, the burst of γH2AX occurred starting at about 12 h (Figure 5B), at which time cells might be entering the S phase. Such information pointed to a possible relationship between PSPC1 and the cell cycle. The following cell cycle analysis indeed revealed that knockdown of PSPC1 disrupted normal cell cycle distribution, with decreased cell numbers in the G1 and S phases, but significantly increased number of cells in the G2/M phase (Figure 6). These results provide evidence that PSPC1 plays a role in cell cycle regulation. Furthermore, cisplatin is known to induce G1/S arrest, during which time damaged DNA can be repaired [49]. However, in PSPC1 knockdown cells, cisplatin-induced G1/S arrest was abolished, and cells continued the cell cycle and entered G2/M (Figure 7). This observation led to the speculation that PSPC1 might be involved in regulation of the G1/S checkpoint.

**Figure 8:**
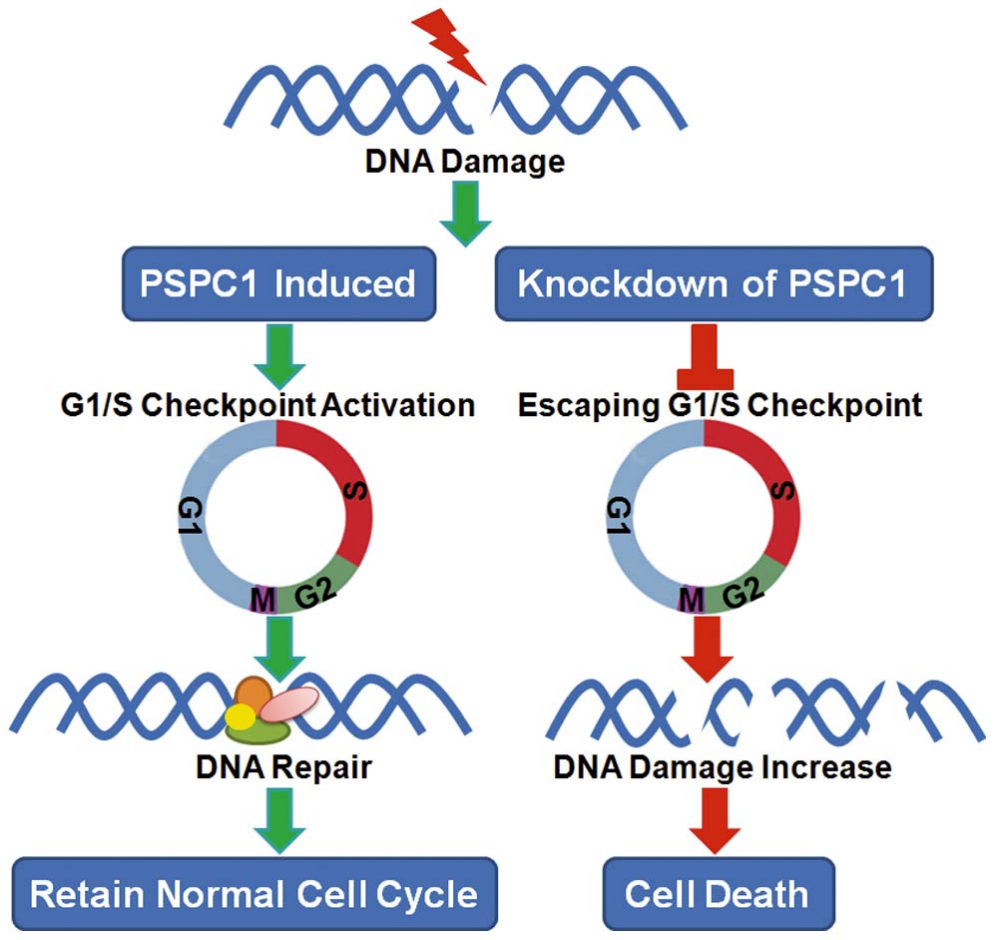
A schematic model for the proposed role of PSPC1 in DDR.

Based on the above results, the following hypothesis is proposed (Figure 8). In normal cells, PSPC1 is required to regulate the G1/S transition. Upon cisplatin exposure, PSPC1 is induced, and coupled with other proteins of the G1/S checkpoint machinery, G1/S arrest is induced, thereby allowing the repair of DNA damage. However, when PSPC1 is knocked down, cisplatin-induced DNA damage cannot activate the appropriate G1/S checkpoint machinery, and cells “slip” through and enter G2/M. As a consequence, cells with unrepaired DNA damage entered mitosis prematurely but cannot complete mitosis, eventually leading to cell death [54]. This could explain the significant cell death observed in PSPC1 knockdown cells.
